# SLC2A9 (GLUT9) mediates urate reabsorption in the mouse kidney

**DOI:** 10.1007/s00424-018-2190-4

**Published:** 2018-08-13

**Authors:** Muriel Auberson, Sophie Stadelmann, Candice Stoudmann, Klaus Seuwen, Robert Koesters, Bernard Thorens, Olivier Bonny

**Affiliations:** 1 Department of Pharmacology and Toxicology, University of Lausanne, 27 rue du Bugnon, 1011 Lausanne, Switzerland; 2 Novartis Institutes for Biomedical Research, CH-4002 Basel, Switzerland; 3 INSERM UMRS 72, UPMC, Tenon Hospital, Paris, France; 4 Centre for Integrative Genomics, University of Lausanne, Lausanne, Switzerland; 5 Service of Nephrology, Department of Medicine, Centre Hospitalier Universitaire Vaudois, Lausanne, Switzerland

**Keywords:** GLUT9, Urate, Uric acid, Lithiasis, SLC2A9

## Abstract

Uric acid (UA) is a metabolite of purine degradation and is involved in gout flairs and kidney stones formation. GLUT9 (SLC2A9) was previously shown to be a urate transporter in vitro. In vivo, humans carrying GLUT9 loss-of-function mutations have familial renal hypouricemia type 2, a condition characterized by hypouricemia, UA renal wasting associated with kidney stones, and an increased propensity to acute renal failure during strenuous exercise. Mice carrying a deletion of GLUT9 in the whole body are hyperuricemic and display a severe nephropathy due to intratubular uric acid precipitation. However, the precise role of GLUT9 in the kidney remains poorly characterized. We developed a mouse model in which GLUT9 was deleted specifically along the whole nephron in a tetracycline-inducible manner (subsequently called kidney-inducible KO or kiKO). The urate/creatinine ratio was increased as early as 4 days after induction of the KO and no GLUT9 protein was visible on kidney extracts. kiKO mice are morphologically identical to their wild-type littermates and had no spontaneous kidney stones. Twenty-four-hour urine collection revealed a major increase of urate urinary excretion rate and of the fractional excretion of urate, with no difference in urate concentration in the plasma. Polyuria was observed, but kiKO mice were still able to concentrate urine after water restriction. KiKO mice displayed lower blood pressure accompanied by an increased heart rate. Overall, these results indicate that GLUT9 is a crucial player in renal handling of urate in vivo and a putative target for uricosuric drugs.

**Electronic supplementary material:**

The online version of this article (10.1007/s00424-018-2190-4) contains supplementary material, which is available to authorized users.

## Introduction

In humans, by contrast to most mammals, uric acid is the end product of purine metabolism, due to silencing mutations acquired over time in the gene coding for uricase, the hepatic enzyme responsible for uric acid degradation into allantoin [41]. Inactivating uricase increases serum uric acid (SUA) levels 5–8-folds and several undemonstrated hypotheses have been proposed to explain why evolution has favored high SUA levels in humans. However, if high SUA brings possible advantages, such as neuro- or immuno-protection [2, 22, 25], it is also increasing the risk for gout and/or kidney stone formation [14, 28]. Indeed, significant amount of uric acid is present in urine and is poorly soluble in this mainly acidic milieu. During the past decades, the prevalence of gout and kidney stones has increased and represents a significant burden for the health system [13, 21, 36].

Urate balance depends on its rate of production and degradation in the liver and on its excretion, mainly by the kidneys. Previous physiological work and genome wide association study analysis have identified several transporters instrumental in transporting urate in both direction (reabsorption and secretion) in the proximal tubule of the kidneys and in keeping serum uric acid concentration constant [8, 12, 19, 20, 23, 40]. One of them, GLUT9, encoded by *SLC2A9*, is responsible for 1.7 to 5.3% of the variation of SUA in humans [19, 40]. GLUT9, originally cloned by homology to the glucose transporter family [11], has been identified as a urate transporter [5, 6]. Its role in urate homeostasis has been validated by the finding of loss-of-function mutations resulting in type 2 familial renal hypouricemia [3, 9, 10, 26, 40]. This inherited disease (OMIM # 612076) is characterized by low levels of SUA mainly due to decreased renal tubular UA reabsorption and high uric acid fractional excretion and is predisposing to exercise-induced acute renal failure and kidney stone formation.

Even if mice have specific ways of handling uric acid (presence of a functional uricase in the liver; different organization of uric acid transporters along the renal tubule), they share several characteristics regarding uric acid metabolism and transport with humans and offer suitable models to study uric acid transport.

GLUT9 is mainly expressed in the liver and the kidneys [4, 17, 29]. In the latter organ, GLUT9 is expressed in the proximal tubule in humans and mice and in the distal tubule of the mouse kidney [31]. Others and our previous work have shown that constitutive deletion of GLUT9 in the whole mice leads to hyperuricemia, hyperuricosuria, and early-onset severe nephropathy [31]. Further, specific deletion of GLUT9 in the liver alone led to hyperuricemia, suggesting a role for GLUT9 in transporting UA into the hepatocytes and making it available for degradation by uricase [31]. Recently, the role of GLUT9 in the intestine has been unraveled by the knock-out of this transporter in enterocytes [7].

However, the role of GLUT9 in the kidney still remains elusive. We have thus generated a kidney-inducible knock-out mouse model for GLUT9. This allowed us to reveal an important role for renal GLUT9 in uric acid homeostasis.

## Materials and methods

### Materials

Products were purchase from Sigma-Aldrich (St. Louis, MO) unless otherwise stated.

### Animals

All animal studies were approved by the state veterinarian Office (Office vétérinaire cantonal, Canton de Vaud, Switzerland). All breeding and cohort colonies were hosted in our animal facility under approved protocols. Mice were housed four to five per cage, with free access to water and food, in a temperature and humidity-controlled room with an automatic 12/12-h light/dark cycle. Animals were fed a standard mouse diet (#3800) from KLIBA (Kaiseraugst, Switzerland).

### Generation of inducible kidney-specific knock-out animals

In order to obtain an inducible cre recombination specifically in the tubular cells of the kidney, a tetracycline-ON system was used. Mice with floxed *Glut9* allele [31] were bred with *Pax8*-rtTA/LC-1 cre-transgenic mice in a C57BL6/N background [38]. Females and males of 6–8-week triple-transgenic mice and their littermate controls (either with *Pax8*-rtTA or LC-1 cre transgene alone) were treated with 2 mg/ml doxycycline in 2% sucrose drinking water for 14 days in order to induce recombination. Fresh spot urine samples were obtained during doxycycline induction and analysis of urate-over-creatinine ratio was performed. Recombination was assessed by PCR using the following primer for *Glut9a*: *mGlut9a*F (GGAGCTTGCTTTAGCTTCCC) and *mGlut9a*R (TGG ACC AAG GCA GGG ACA A), and for *Glut9b*: *mGlut9b*F (AAC TCC GCA GAA ACC AAG GAA AGC) and *mGlut9b*R (ACCCATGATGAACCGTCCCA). PCR-generated fragments were 664 and 613 bp for *Glut9a* WT and kiKO respectively and 481 and 430 bp for *Glut9b* WT and kiKO respectively. Unless otherwise specified, mice were used 4 months after the end of doxycycline induction.

### Genotyping

Genotypes were determined by PCR on total genomic DNA extracted from ear biopsy by NaOH digestion and using the following primers: *Glut9*F (CTG TCC AGA TGT TGT CTA GG), *Glut9*R (GTT ATG ATG CAG GAG CTT AGC), LC1-Cre-F (TCG CTG CAT TAC CGG TCG ATG C), LC1-Cre-R (CCA TGA GTG AAC GAA CCT GGT CG), *Pax8*-F (CCA TGT CTA GAC TGG ACA AGA), *Pax8*-R (CTC CAG GCC ACA TAT GAT TAG). PCR reactions were carried out on a PeqStar 2x Thermal Cycler (PeqLab Biotechnologie, Erlangen, Germany) using GoTaq DNA Polymerase (Promega Corporation, Madison, WI). PCR protocol was 5 min at 95 °C followed by 35 cycles (1 min at 95 °C, 1 min at 60 °C, and 2 min at 72 °C) and by 10 min at 72 °C. This generated a fragment of 361 bp for GLUT9 wild-type allele and of 460 bp for the floxed allele. *Pax8*-rtTA and LC1-cre transgene were detected by fragments of 650 and 430 bp respectively.

### Metabolic cages, blood, and urinary analysis

The mice were placed in individual metabolic cage (Tecniplast, Buguggiate, Italy) for 2 days in order to get used to the new environment, before two cycles of 24-h urine collection. Food and water intake were measured, and the 24-h collected feces were weighed. Determination of excretion rate was calculated as the concentration of a given substance in the volume of the 24-h urine. Body weight was also determined. Plasma and urine chemistry were analyzed using a Roche/Hitachi 902 robot system (Roche, Mannheim, Germany). Osmolality was measured with a vapor pressure osmometer (Vapro 5520, Wescor, South Logan, USA).

### Microdissection of nephron segments

After deep anesthesia by intraperitoneal injection by Ketanarkon (Streuli, Pharma AG, Switzerland; 100 μg/g body weight) and Rompun (Bayer, Leverkusen, Germany; 10 μg/g body weight), the left kidney was perfused with DMEM/F12 (1:1, Life Technologies, Carlsbad, USA) supplemented with 40 mg/ml of Liberase Blendzyme 2 (Roche, Switzerland). The kidney was cut in thin pyramids along the corticomedullary axis and incubated 40 min at 37 °C. After washes with DMEM/F12 to stop the digestion, microdissection of glomerulus (glom), proximal convoluted tubule (PCT), proximal straight tubule (PST), medullary thick ascending limb (TAL), distal convoluted tubule and cortical connecting tubule (DCT-CNT), or cortical collecting duct (CCD) was performed in DMEM/F12 (1:1). Tubular length was measured with an ocular micrometer, and pools of 10–20 microdissected tubules covering a total tubular length of ∼ 10 mm/pool were transferred in sample buffer for Western blot analysis.

### Osmolality measurements in kidney fragments

Under deep anesthesia and after cervical dislocation, kidneys were removed and cut in slices and small parts of cortex and medulla were dissected and weighted. Fifty microliters of water was added to the samples. After centrifugation 10 min at 12,000 rpm, the supernatants were collected and osmolality of the sample was measured and normalized to the weight of the fragment.

### Immunoblotting

For protein extraction, half kidneys were homogenized in RIPA buffer (Tris pH 7.2 50 mM, NaCl 150 mM, NP40 1%, SDS 0.1%, Na-deoxycholate 0.5% with proteases inhibitor) using TissueLyser (Qiagen, Hilden, Germany). After centrifugation at 12,000 rpm for 15 min, the supernatants were collected and total protein concentration was determined using a BCA protein assay kit (Pierce, Rockford, IL). Equal amounts of protein were separated on 10% SDS polyacrylamide gels and blotted onto nitrocellulose membrane (Whatman, Dassel, Germany). Primary antibodies used were GLUT9 (1:500, [31]), AQP2 (1:500, kindly provided by Prof. J. Loffing, Institute of Anatomy, University of Zurich), NCX1 (1:1000, [30]), and actin (1:500, Sigma, St-Louis, USA). Secondary anti-rabbit and anti-mouse antibody (1:10,000, Amersham Biosciences, Buckinghamshire, UK) were detected by chemiluminescence (Super Signal West Pico, Thermo Scientific, Rockford, USA).

### Real-time PCR

Half kidneys were sampled and homogenized in a TRI Reagent solution (Ambion, Austin, USA) followed by an extraction with 1-bromo-3-chloropropane reagent (BCP, Molecular Research Center, Cincinnati, USA) and an isopropanol precipitation. RNA (1 μg) was reverse transcribed using a PrimeScript RT reagent kit (Takara Biotechnology, Otsu, Japan) according to manufacturer’s guidelines. TaqMan Gene Expression Assays (Applied Biosystems, USA) were used to detect *Urat1* (Mm01236822_m1), *Mrp4* (Mm01226381_m1), *Abcg2* (Mm00496364_m1), *Npt1* (Mm00436577_m1), *Npt4* (Mm00506321_m1), *Oat1* (Mm00456258_m1), *Oat3* (Mm00459534_m1), *Oat10* (Mm00506015_g1), uricase (*Uox*, Mm00447661_m1), xanthine dehydrogenase (*Xdh*, Mmoo442110_m1), *V2r* (Mm01193534_g1), *Aqp2* (Mm00437575_m1), *Glut9a* (Mm01211146_m1), *Glut9b* (Mm00455117_m1), and *Actb* (actin, beta, VIC/MGB Probe, Primer Limited).

For quantification of the recombination of *Glut9*, primers targeting exon 4 which is deleted after cre recombination were designed (*Glut9* ex4 F: TTG GGA GGA AGT CCA CAT TGC TGG, *Glut9* ex4 R: TCC ATC CAC ACC CAT GAT GAA CCG) and used with primers for *Actb*: *Actb* F (GTC CAC CTT CCA GCA GAT GT) and *Actb* R (AGT CCG CCT AGA AGC ACT TGC). Quantitative real-time PCRs were carried out on an ABI PRISM 7500 equipment (Applied Biosystems, Carlsbad, USA) in triplicate for each sample, either with TaqMan Universal PCR Master Mix (Applied Biosystems) or SYB green PCR Master Mix (Applied Biosystems) in a final volume of 20 μl.

The relative expression of each gene was calculated using the comparative 2^[−ΔΔCT]^ method [42], normalized to *Actb*. Data are represented as relative fold-change compared to control mice.

### Fixation, tissue processing, and immunofluorescence

Mice were anesthetized by intraperitoneal injection of Ketanarkon (Streuli, Pharma AG, Switzerland; 100 μg/g of body weight) and Rompun (Bayer, Leverkusen, Germany; 10 μg/g of body weight).The left kidney was perfused via the abdominal aorta with paraformaldehyde 4% in PBS (137-mM NaCl, 2.7-mM KCl, 0.9-mM KH_2_PO_4_, 6.4-mM NaH_2_PO_4_, pH 7.4). Kidneys were incubated in 30% sucrose in PBS for at least 24 h before being embedded in Tissue-Tek OCT compound (Sakura Finetek, Netherland) and frozen. Eight-micrometer thick cryosections were incubated 1 h with blocking buffer (NP-40 0.5%, BSA 2%, normal goat serum (NGS) 3% in PBS) at room temperature. Incubation with primary antibodies diluted in blocking buffer without NGS was performed overnight at 4 °C. Used primary antibodies were GLUT9 (1:500, [31]), NCX1 (1:1000, [30]), and AQP2 (1:500, kindly provided by Prof. J. Loffing, Institute of Anatomy, University of Zurich). After washing three times with PBS, sections were incubated 1 h at room temperature with secondary antibodies AlexaFluor488 (1:2000 diluted in blocking buffer without BSA, Invitrogen, Carlsbad, USA) and then washed four times with PBS. The sections were then mounted using Fluoromount-G mounting medium (Southern Biotech, Birmingham, USA). Fluorescent images were visualized using a laser scanning confocal microscope (Leica SP5 AOBS Confocal Microscope).

### Telemetry

Measurements of blood pressure were performed by telemetry using a specific transducer for mice (Data Sciences International, St. Paul, USA). Under deep anesthesia, catheter was introduced into the left carotid and the transducer was placed in the abdominal cavity. Mice were placed in the recording cages individually, but were keeping olfactory and visual contact with conspecifics and were able to run freely. At least 14 days after surgery, the device was switched on and the mouse cage was placed on an antenna. The signal was then processed by a software and translated into pressure curve (Dataquest A.R.T.™ acquisition and analysis system (Data Sciences International, St. Paul, USA)).

### Statistical analyses

Results are presented as means ± SD. Statistical analyses were performed using GraphPad Prism 6.0 software (GraphPad Software Inc.). Comparison between groups was performed using one-way ANOVA test followed by Bonferoni post hoc test. Student’s *t* test (2-tailed) for unpaired data was used. *p* values < 0.05 were considered as statistically significant.

## Results

### GLUT9 localization in the mouse kidney

GLUT9 precise localization in the kidney is subject to controversy as several reports have shown various tubular expression and membrane sorting. Indeed, in transfected MDCK cells, murine GLUT9 was observed at the basolateral membrane [17], whereas immunohistochemical staining of GLUT9 on mouse kidney sections showed both apical and basolateral staining [31].

**Fig. 1:**
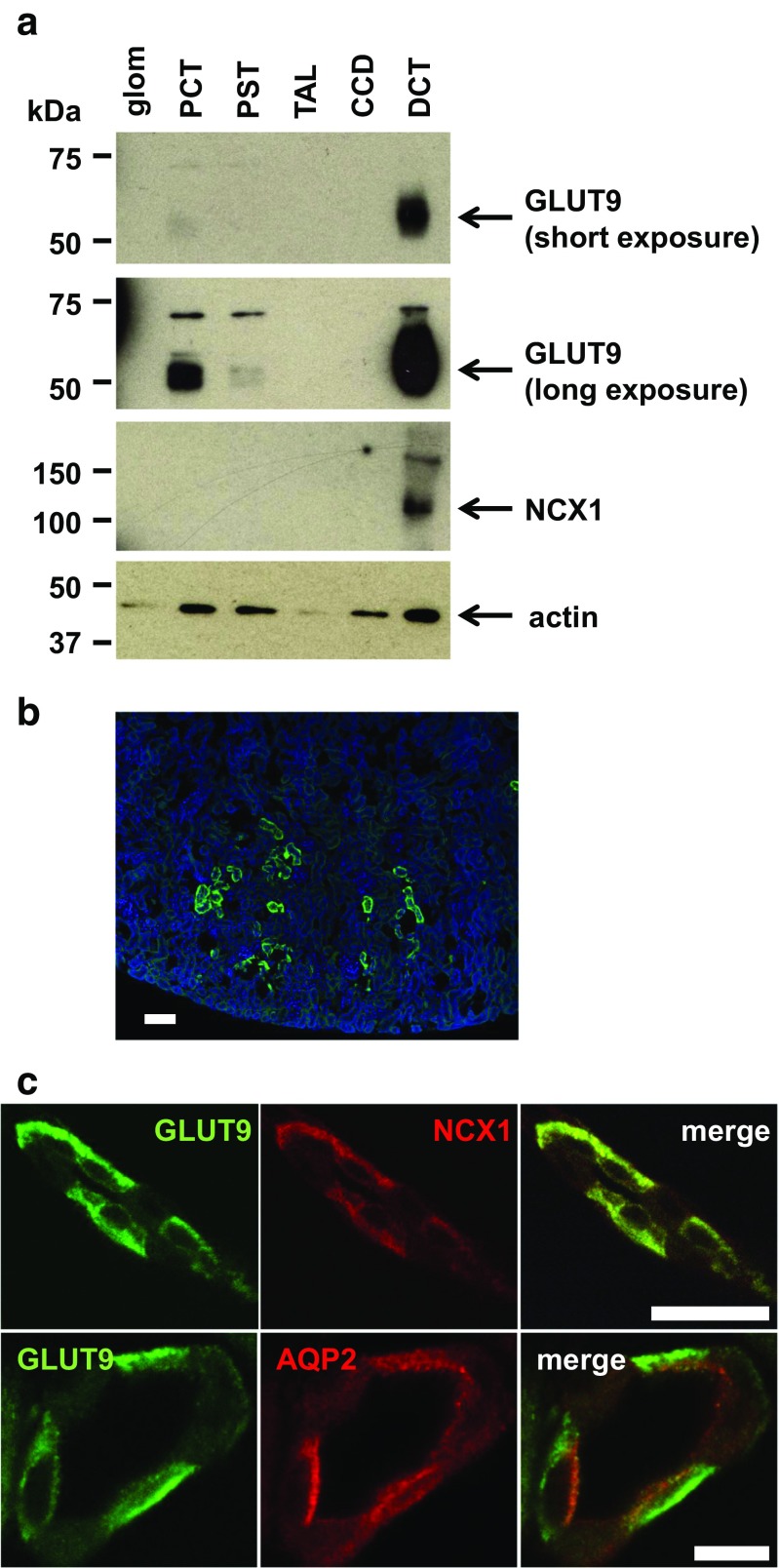
GLUT9 is mainly expressed at the basolateral membrane of the DCT and slightly in the PCT. **a** Western blot of microdissected tubules from wild-type mice. GLUT9 is strongly detectable in the DCT. Some expression is also visible in the PCT after a longer exposure. NCX1 is used as positive control for the accuracy of the DCT microdissection. Protein loading can be evaluated by actin quantification. **b** Immunostaining of GLUT9 on wild-type kidney section. GLUT9 signal is restricted to cortical distal convoluted tubules (scale bar: 100 μm). **c** Co-immunostaining of GLUT9 and NCX1, and of GLUT9 and AQP2 on wild-type kidney sections. Both GLUT9 and NCX1 signals are co-localizing at the basolateral membrane of the DCT. There is no co-localization of GLUT9 and AQP2 (scale bar: 10 μm)

We took advantage of a newly in-house produced anti-GLUT9 antibody to analyze GLUT9 expression in vivo (Supplementary Fig. S1). First, we microdissected different parts of the nephron (glomerulus, PCT, PST, TAL, DCT, and CCD) of wild-type mice and looked for GLUT9 expression. A strong signal appearing at molecular weight 55 kDa was readily visible in the distal convoluted tubule (DCT), and only after longer exposure in the proximal convoluted tubule (PCT) as well (Fig. 1a). Immunostaining of GLUT9 revealed staining in the cortical kidney (Fig. 1b). More specifically, we found strong basolateral GLUT9 staining in the DCT (Fig. 1c), as indicated by perfect co-localization of GLUT9 with NCX1, the basolateral calcium-sodium exchanger type 1 expressed in this segment [24]. Moreover, no co-localization could be detected with AQP2 (Fig. 1c), an apical water channel expressed in the same cells. No staining for GLUT9 in proximal tubules was visible on kidney sections, probably due to the weak GLUT9 expression, as anticipated from the Western blot (Fig. 1a). This data shows strong evidence for basolateral expression of GLTU9 in the mouse DCT and weaker expression in the proximal tubule.

### Doxycycline-inducible GLUT9 deletion in the kidney: molecular analysis

**Fig. 2:**
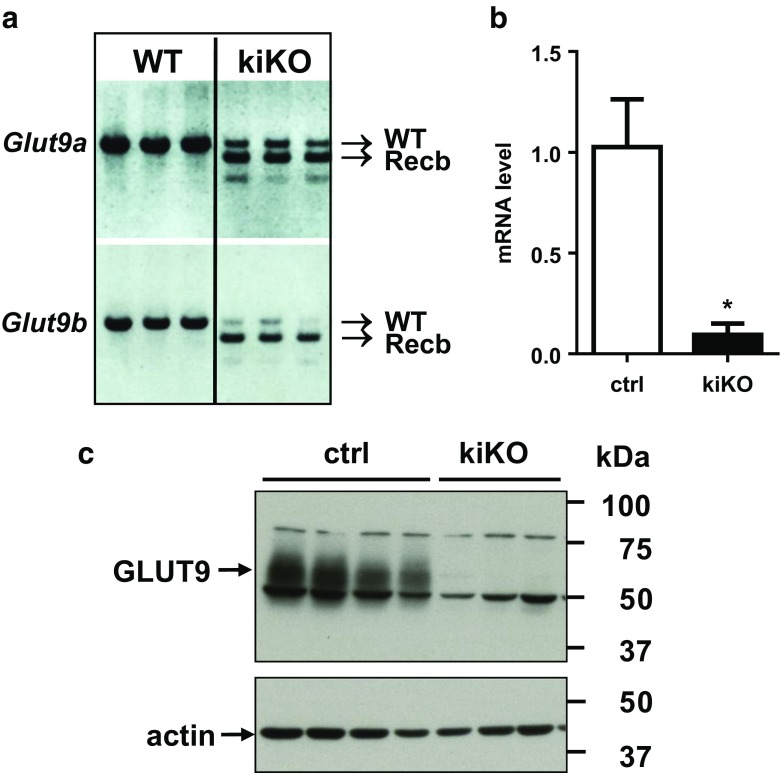
Doxycycline-induced deletion of GLUT9 in the kidney: molecular analysis. **a** PCR on cDNA obtained 4 months after doxycycline induction from control and kiKO mouse kidneys. Recombination of both *Glut9a* and *b* isoforms is observed in the kidney of kiKO mice (*n* = 3). **b** Relative abundance of *Glut9* transcript from total kidney 4 months after doxycycline induction, as measured by quantitative real-time PCR. Values are means ± SD relative to control (*n* = 10, *p < 0.05, by Student’s *t* test). **c** Renal GLUT9 protein expression levels in control and kiKO mice by Western blot 4 months after doxycycline induction. No GLUT9 protein is detectable in the kidney of kiKO mice (*n* = 3–4). Protein loading was evaluated by actin

In order to determine the specific role of GLUT9 in the kidney, an inducible kidney-specific *Glut9* knock-out mouse model (hereafter called kiKO) was generated by using mice carrying, on the one hand, the floxed GLUT9 allele [31] and, on the other hand, the *Pax8*-rtTA/LC-1 cre recombinase [38]. *Glut9*^flox/flox^/*Pax8*-rtTA/LC1 (kiKO) mice and littermate controls (*Glut9*^flox/flox^/*Pax8*-rtTA or *Glut9*^flox/flox^/LC1, called controls hereafter) were treated with doxycycline added to drinking water for 14 days. Recombination of *Glut9*a and *Glut9*b isoforms was observed at least 6 days after doxycycline treatment (Fig. S2). Four months after the end of the doxycycline treatment, mice still exhibited recombination of *Glut9* in the kidney (Fig. 2a) with only 7.4 ± 2.7% of residual *Glut9* RNA expression (Fig. 2b). Renal GLUT9 protein expression level confirmed the absence of GLUT9 protein in the whole kidney extract of kiKO mice compared to controls (Fig. 2c). In the liver, Traykova-Brauch et al. [38] have shown a partial *Pax8*-rtTA-mediated recombination in some periportal hepatocytes. We checked whether *Glut9* recombination also occurred in the liver of kiKO mice. During doxycycline induction, a partial recombination of *Glut9* could be observed in the liver of the mice (Fig. S3a). This recombination was still observed 4 months after induction, with decreased liver GLUT9 expression of 68.4 ± 15.6% and 61.6 ± 29.3% for respectively mRNA and protein (Fig. S3b, c). Of note, females were less affected by the recombination in the liver than males. As additional control for organ specific deletion, we also looked at putative GLUT9 deletion along the intestine. Fig. S4a, b indicates an absence of *Glut9* recombination in the ileum and in the colon.

### Doxycycline-inducible Glut9 deletion in the kidney: functional analysis

**Fig. 3:**
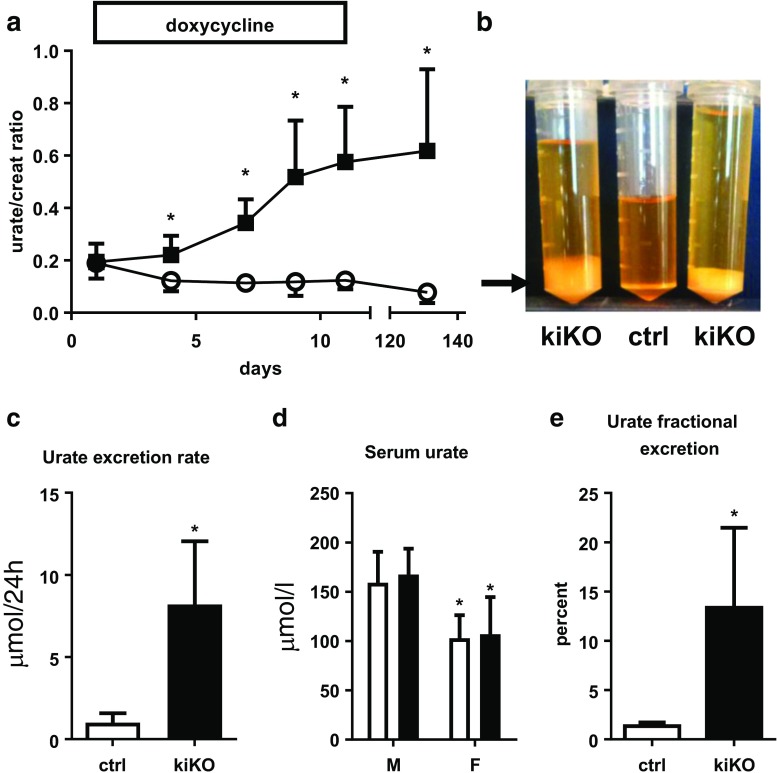
Doxycycline-induced deletion of GLUT9 in the kidney: functional analysis. **a** Time-course of the urate/creatinine ratio from spot urine after doxycycline induction (starting at day 0). Four days after the induction, the urate/creatinine ratio is significantly increased in kiKO mice compared to control. Four months after the induction by the doxycycline, the difference between control and kiKO mice is still present. Values are means ± SD (*n* = 6, *p < 0.05 by Student’s *t* test). **b** Twenty-four-hour urine collection of kiKO mice presents an important white deposit (arrow) when kept at room temperature. This deposit is made of uric acid crystals (not shown) and is absent in control urine. The 24-h volume of kiKO mice urine is higher than the volume of control urine and accordingly, urine is more diluted. **c** Measurement of 24-h urate excretion rate. Urate excretion rate for kiKO mice is higher than for controls. Values are means ± SD (*n* = 8, *p < 0.05). **d** SUA analysis. There is no difference between control (white bars) and kiKO (black bars) mice regarding plasma concentration of urate. Males had higher urate concentration in the plasma than females. Values are means ± SD (*n* = 8, *p < 0.05). **e** Fractional excretion of urate (FE urate). A significantly higher FE urate was measured in kiKO mice compared to controls. Values are means ± SD (*n* = 8, *p < 0.05)

The time-course of the effect of doxycycline-mediated *Glut9* deletion in the kidney was monitored by measurement of the urate-over-creatinine ratio (urate/creat) on fresh urine. A significant increase of the urate/creat ratio was observed already 4 days after induction (Fig. 3a), reaching a peak of 11 days after the beginning of the doxycycline treatment, with an increase of 2.7 ± 1.2 fold compared to control. Sodium-over-creatinine ratio was unchanged (Fig. S5a). One month after doxycycline induction, 24-h urine collection was performed in metabolic cages. Immediate void urines from kiKO and wild-type mice were clear, whereas a white deposit was apparent in the urine of kiKO mice left at room temperature (arrow, Fig. 3b). An increase of urine volume per 24 h was noted in kiKO mice (Fig. 3b). Urate excretion rate was markedly increased in kiKO compared to control mice (7.08 ± 2.27 fold increases, Fig. 3c). No difference was observed regarding daily food or water intakes, and the body weight was similar between control and kiKO mice (not shown). The urinary pH was unchanged between the two genotypes (6.17 ± 0.46 for control and 6.21 ± 0.30 for kiKO). SUA was significantly different between male and female mice, but no change was detected when comparing SUA between control and kiKO mice (Fig. 3d). Consequently, an increase of the urate fractional excretion was observed in kiKO mice, for both, male and female mice (Fig. 3e). The GLUT9 full body knock-out mouse model presented with a severe nephropathy including hydronephrosis, cortical fibrosis, and renal insufficiency [31]. By contrast, histomorphologic analysis of kiKO kidneys did not show any change compared to controls (Fig. S5b, c). No fibrosis and no inflammation were detected, neither by quantification of markers by qPCR (Fig. S6a), nor by Masson’s trichrome staining (Fig. S6b). Moreover, kiKO mice plasma creatinine levels were similar to control mice (17.3 ± 3.2 μM in kiKO versus 17.5 ± 4.7 μM in control), indicating an absence of renal insufficiency.

### Polyuria and water homeostasis in kiKO mice

**Fig. 4:**
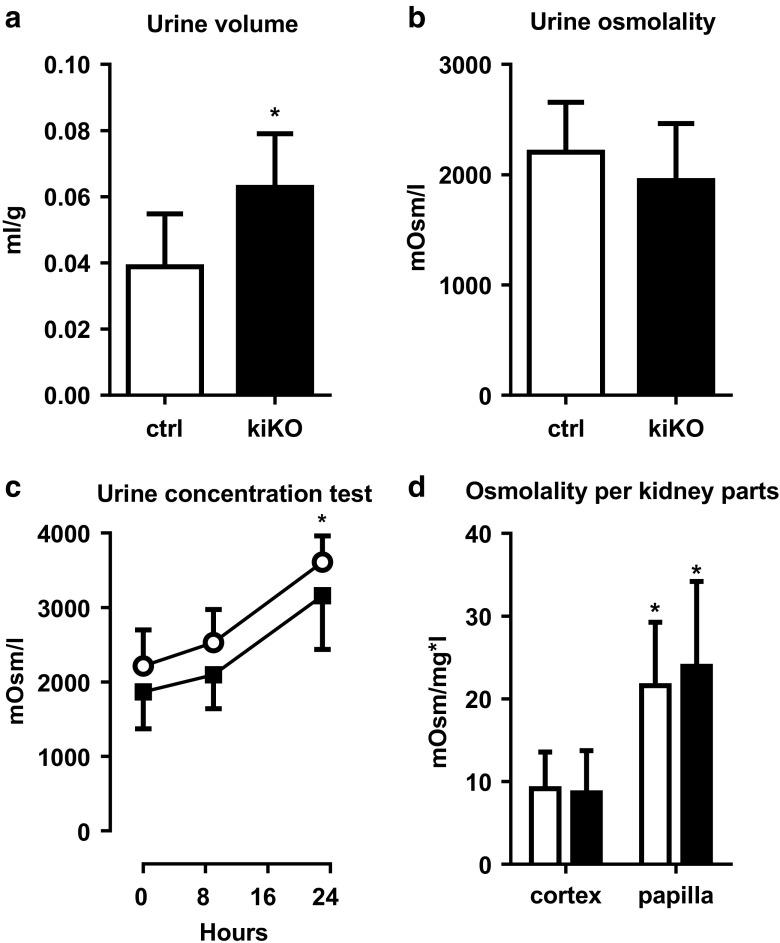
More diluted urine in the hyperuricosuric kiKO mice. **a** KiKO mice display an increase urine volume compared to control mice. Values are means ± SD (*n* = 16, *p < 0.05). **b** Urine osmolality is not changed between control and kiKO mice. Values are means ± SD (*n* = 23). **c** Urine concentration test. After 9 and 23 h of water deprivation, both control and kiKO mice are able to concentrate their urine the same way, with significantly increased urine osmolality compared to baseline, but no difference between the two genotypes. Values are means ± SD (*n* = 10). d. Measurement of osmolality in the cortex and the papilla of control (white bars) and kiKO (black bars) mice did not show any difference between both genotypes. A significant increase of osmolality is measured in the papilla by comparison with the cortex, for both control and kiKO mice. Data are expressed as mOsm/l per mg of renal tissue. Values are means ± SD (*n* = 20, *p < 0.05)

**Fig. 5:**
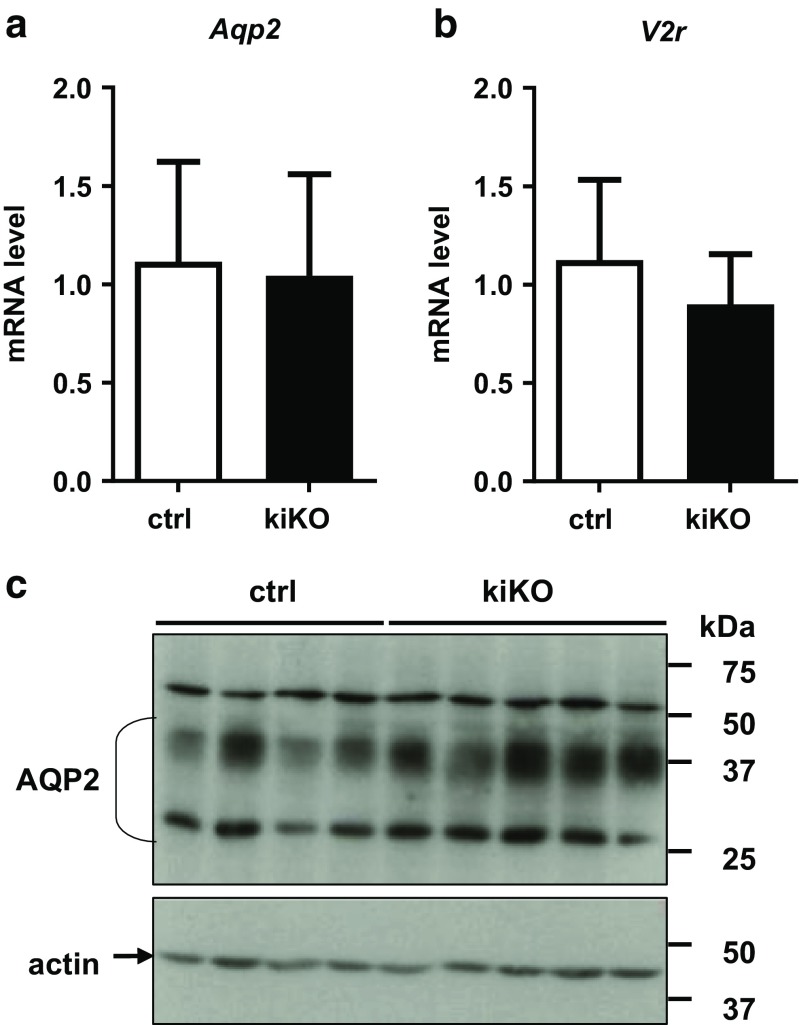
Expression level of AQP2 and V2R in the kidney. No difference in the *Aqp2* (**a**) and *V2r* (**b**) expression level was observed between control and kiKO mice by qPCR. Values are means ± SD (*n* = 19 for *Aqp2* and *n* = 9 for *V2r*). **c** AQP2 protein expression level in control and kiKO mice. Values are means ± SD (*n* = 3 to 5)

A 20% increase of the urine volume was observed in the kiKO mice, with a corresponding trend toward decreased urine osmolality compared to control mice (Fig. 4a, b). The urinary concentrating capacity of the kiKO mice was challenged by a water deprivation test. Mice of the two genotypes were able to concentrate urine over time (Fig. 4c). Disturbed urine concentration was further explored. Parts of the cortex and papilla of control and kiKO mice were isolated and osmolality was measured. Significant and expected increase of the osmolality was observed in the papilla compared to the cortical part, but no difference in the corticomedullary osmotic gradient was observed between control and kiKO mouse kidneys (Fig. 4d). Two important effectors of water handling by the kidney are aquaporin-2 (AQP2) and the vasopressin receptor type 2 (V2R). By qPCR, *Aqp2* and *V2r* mRNA expressions in whole kidney extract were similar in control and kiKO animals (Fig. 5a, b) under standard conditions and after water deprivation (Fig. S7). AQP2 protein expression was further analyzed by Western blot. No significant difference of AQP2 protein expression was noticed between control and kiKO kidneys (Fig. 5c).

We previously showed in the full body GLUT9 KO mouse model that polyuria was accompanied by urine acidification evoking similar processes found in the hypercalciuric TRPV5 knock-out mice and thought to be mediated by the calcium sensing receptor [15]. We thus tested whether the calcium sensing receptor (CaSR) could be triggered by high urinary uric acid levels in the presence of calcium and could mediate polyuria. We used HEK cells stably expressing CaSR and we exposed them to increasing concentrations of calcium and urate. As illustrated in Fig. S8, no additional activation of CaSR was observed in presence of urate. Therefore, CaSR seems unlikely to play any direct role in modulating urine acidification and dilution in these mice.

### Compensatory mechanisms

As presented in Fig. 3c, kiKO mice presented an 8-fold increase of urate excretion rate compared to control mice, but a normal SUA (Fig. 3d), even though hypouricemia was expected. We thus looked for possible compensatory mechanisms that may explain unchanged SUA.

**Fig. 6:**
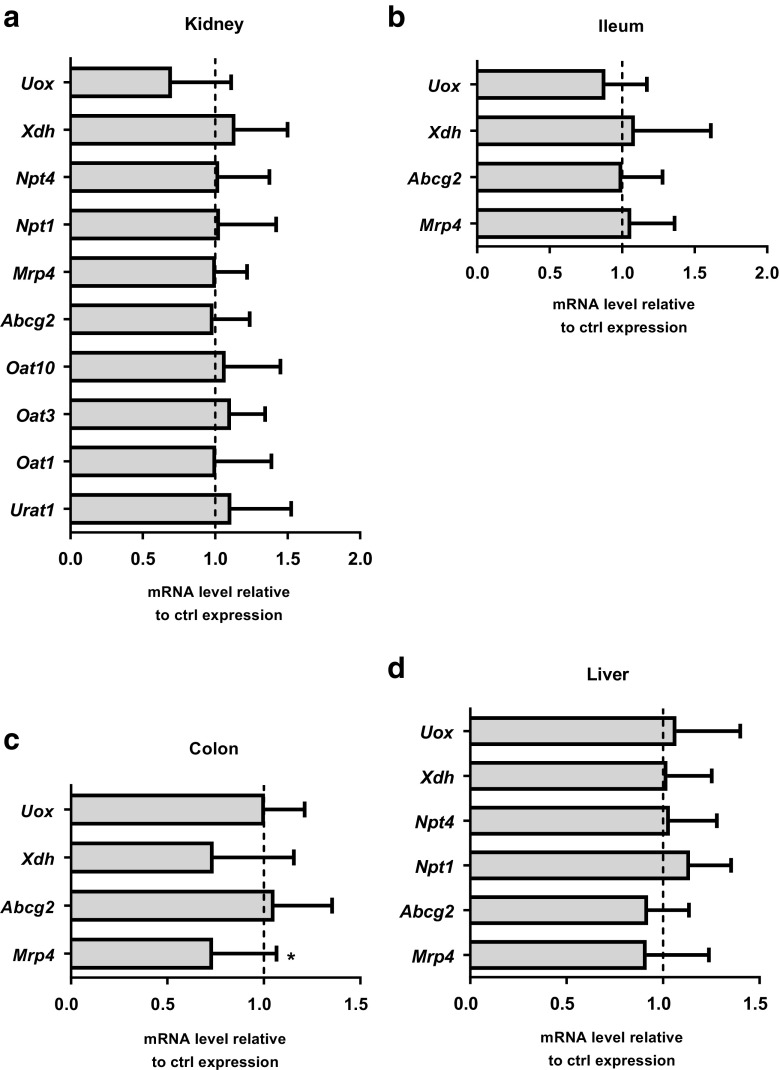
Possible compensatory mechanisms. qPCR analysis of the relative abundance of several known urate transporters and enzymes involved in urate metabolism in the kidney (**a**), the ileum (**b**), the colon (**c**), and the liver (**d**). Data are normalized to control expression. Values > 1 indicate higher expression in kiKO mice compared to controls. Values are means ± SD (*n* = 5, *p < 0.05)

Several organs participate in urate homeostasis, namely the kidney, the intestinal tract, and the liver. Besides GLUT9, many transporters are involved in the handling of urate, such as URAT1, ABCG2, MRP4, OAT1, OAT3, and OAT10 [37]. Moreover, the metabolism of urate in mice is mainly due to two enzymes: the xanthine oxidase, which mediates the conversion of xanthine into urate [1], and the uricase, which catabolize urate into allantoin [41]. Expression analysis of these different transporters and enzymes was performed by qPCR on cDNA extracted from the kidney, liver, ileum, and colon of control and kiKO mice. Results are illustrated in Fig. 6 and show a down-regulation of the expression of *Mrp4* in colon (Fig. 6c), but otherwise, no other compensatory mechanisms could be detected.

### Blood pressure analysis in kiKO mice

**Fig. 7:**
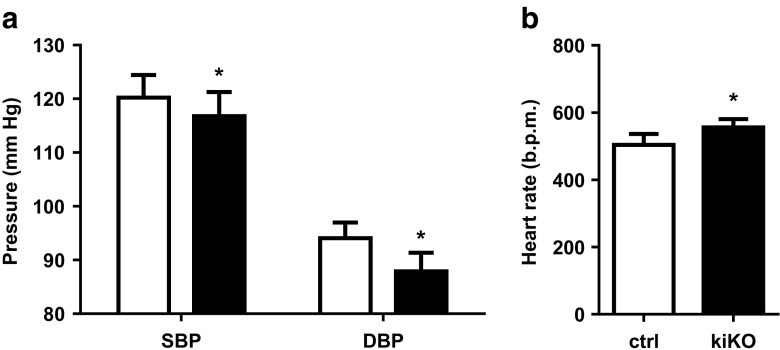
kiKO mice have lower blood pressure and higher heart rate. **a** Measurement of blood pressure in control (white bars) and kiKO (black bars) mice. A decrease of systolic (SBP) and diastolic (DBP) blood pressure is observed in kiKO mice. Values are means ± SD (*n* = 6, *p < 0.05). **b** Heart rate (in beats per minutes, b.p.m.) was higher in kiKO mice compared to control mice. Values are means ± SD (*n* = 6, *p < 0.05)

Studies have shown that moderate hyperuricemia may cause hypertension in rats [27], findings that have been recently challenged [32]. We took advantage of the kiKO mouse model to study the impact of increased uric acid excretion on blood pressure. By telemetry, diastolic and systolic blood pressures were measured for 1 week. Results in Fig. 7a showed a slight decrease of both diastolic and systolic blood pressure. At the same time, an increase of the heart rate was observed (Fig. 7b).

## Discussion

This study identifies GLUT9 as a critical player in renal urate reabsorption in mice. We showed that loss of expression of GLUT9 along the renal tubules induces a significant leak of uric acid in the urine accompanied by an increase in urine volume. The renal architecture and the filtration capacity were preserved in these mice.

This model displays some peculiarities compared to previous GLUT9 KO mice models, especially regarding urine dilution and SUA levels. GLUT9 constitutive systemic KO mice developed moderate hyperuricemia, massive hyperuricosuria, low urine pH, inability to concentrate urine, and an early-onset severe nephropathy with intratubular obstructive uric acid crystals, tubulointerstitial inflammation, fibrosis, and progressive renal insufficiency. Liver-specific inactivation of GLUT9 in adult mice conducted to strong elevation of SUA, hyperuricosuria, lower urine pH, and blunted capability of urine concentration, but no structural abnormality of the kidney was observed. In this study, renal tubular inactivation of GLUT9 led to a milder phenotype, with moderate hyperuricosuria, but with no change in SUA, urine pH, or renal structure. A moderate increase in urine volume without alteration of the urine-concentration ability was also noted. We looked in more details at the correlation between high uricosuria and urine dilution that was observed in all three GLUT9 KO models [31]. Compared to the systemic and the liver-specific GLUT9 KO mouse models, deletion of GLUT9 along the renal tubule did not affect the overall concentration capacity of the kidney, as illustrated by a water restriction test. We measured whether the osmotic gradient along the corticomedullary axis could be affected and lead to concentrating defect, but it was preserved in kiKO mice. We did not observe any difference in the expression level of the vasopressin receptor V2R or of the aquaporin 2, even after water restriction. Finally, we looked for a possible direct role of the calcium sensing receptor in the observed urate-dependent urine dilution. Indeed, the calcium sensing receptor—or a similar mechanism—has been proposed to mediate urine dilution in hypercalciuria [35] and to play a protective role against intratubular crystal formation. We tested whether the CaSR may mediate urate-dependent signaling, but could not show any influence of increasing urate concentration on CaSR-dependent signaling. This suggests that other sensing mechanisms might be involved in this process. Overall, we could not identify a specific mechanism that may explain the increased urine dilution in this mouse model. However, the methods used might not be sensitive enough to detect small changes as observed in this model compared to other GLUT9 KO models.

KiKO mice have normal serum uric acid levels despite a significant loss of uric acid in the urine. This constitutes one major difference with humans suffering of GLUT9 loss-of-function mutations who display severe hypouricemia. Several possible explanations could be brought forward here. First, by contrast to humans, mice express uricase and the presence of this enzyme already reduces SUA to minimal levels that may not allow further decrease. Studying the kidney-specific role of GLUT9 in an UOX knock-out background would be a way of circumventing this limitation [16]. Second, and as observed in the initial description of the model [38], the PAX8 promoter that drives the cre recombinase expression leads to some recombination in the liver (Fig. S3). We previously showed that homozygous—but not heterozygous—deletion of GLUT9 in the mouse liver by using the albumin-driven cre recombinase led to strong increase of SUA [31, 33]. Inability of urate to enter hepatocytes through GLUT9 and be degraded by uricase is thought to account for this elevation of SUA. In the present model, the small deletion of GLUT9 in the liver may be enough to blunt the expected hypouricemia in these mice. Finally, compensatory mechanisms by other urate transporters in the kidneys or in other organs may maintain SUA constant in this mouse model despite the induced renal leak. We studied the expression levels of the main transporters and proteins involved in the maintenance of uric acid homeostasis. The only difference between control and kiKO mice is a decreased expression of the gene coding for MRP4 in the colon of kiKO mice. Analysis of MRP4 protein expression levels would be needed before drawing any conclusion, but we can extrapolate that a decreased secretion of uric acid in the colon would be expected if the decreased expression of MRP4 would be confirmed at the protein level. However, if transport of urate was shown in cells transfected with MRP4 [39], no physiological evidence of its role in uric acid homeostasis in vivo exists so far, especially of its role in the colon. Overall, we could not identify compensation mechanisms in kiKO mice, but further studies are needed as transcriptional regulation of these transporters has not been explored.

An interesting finding of this report relates to the decreased blood pressure observed in kiKO mice. Uric acid is pointed as a key player in the maintenance of blood pressure and hyperuricemia is often associated with hypertension. However, causality is debated and Preitner et al. have recently shown, in an elegant study, that no correlation between SUA and blood pressure is observed when SUA is increased stepwise [33]. Here, we found that the renal leak of uric acid lowers blood pressure without changes in SUA. The presence of increased heart rate is however more suggestive of a slight state of dehydration due to the increased urine volume. We are not providing data however to support this working hypothesis and possibility of a direct effect of GLUT9 function in the proximal or distal tubules remains open. Of note, blood pressure analyses were carried out only in males and the conclusion may not apply to females.

As previously related, the phenotype observed in these mice is different from traits identified in humans suffering from familial hypouricemia type 2 for several reasons: presence of a functional hepatic uricase; debated role of GLUT9 in the human liver; and debated localization and sorting of GLUT9 isoforms in the different segments of the kidneys. Here, we confirmed unambiguously that in the mouse, GLUT9 is mainly located in the distal convoluted tubule [17], with lower expression in the proximal convoluted tubule, while this carrier is located exclusively in the proximal tubules in humans [4]. The sorting of mouse and human GLUT9 isoforms in cells seems also to differ. Mouse GLUT9 isoforms are expressed at the basolateral side of MDCK transfected cells [17], a pattern compatible with our own data (Fig. 1c). For human isoforms, some controversies exist. The long isoform is consistently expressed at the basolateral side of MDCK transfected cells, whereas the short isoform is described either only at the apical side [4] or at both apical and basolateral sides of MDCK transfected cells [18]. Altogether, mice have a unique expression pattern of GLUT9 in the distal convoluted tubule that is of unknown function. Expression at a lower level in the proximal tubule is compatible with a more traditional role in transcellular reabsorption of uric acid in this part of the tubule.

Overall, this work identifies GLUT9 as a critical partner in renal uric acid handling and more precisely in urate reabsorption. It points to a role of GLUT9 in the mouse distal convoluted tubule that remains unknown. Loss-of-function mutations in humans lead to severe hypouricemia, at the level of uricase-expressing species [34]. Mice with renal specific deletion of GLUT9 do not display hypouricemia even in presence of urate leak, but have higher urine volume and lower blood pressure. Extrapolation of this data to humans should be made only with caution as several differences are prominent in the way uric acid is handled in these two species.

## Electronic supplementary material

ESM 1 (PDF 1183 kb)
