# Supplementary material for: SLC2A9 (GLUT9) mediates urate reabsorption in the mouse kidney
Source: Pflugers Arch. 2018 Aug 13;470(12):1739–51. doi: 10.1007/s00424-018-2190-4 (PMC6224025; doi:10.1007/s00424-018-2190-4)
Supplement: Supplementary file 1 — (PDF 1183 kb) [file 424_2018_2190_MOESM1_ESM.pdf]

SUPPLEMENTARY MATERIAL

GLUT9 mediates urate reabsorption in the mouse kidney

Auberson Muriel, Stadelmann Sophie, Stoudmann Candice, Seuwen Klaus, Koesters Robert, Thorens Bernard, and Bonny Olivier

Corresponding author: Dr. Olivier Bonny, Department of Pharmacology and Toxicology, University of Lausanne, 27 rue du Bugnon, 1011 Lausanne, Switzerland. Email: Olivier.Bonny@unil.ch, phone: +41216925364, fax: +41216925355

Supplementary methods

Histology

Cryosections, prepared as already described, were stained with hematoxylin and eosin according to standard histological protocol. Paraffin section were stained following Masson's trichrome protocol.

Real-time PCR

TaqMan Gene Expression Assays (Applied Biosystems, USA) were used to detect fibrosis markers: *Vimentin* (Mm01333430_m1), *Col1a1* (Mm00801666_g1), *Col3a1* (Mm00802300_m1) and *Fibronectin* (Mm01256744_m1). Primers used to detect inflammation markers were T*gfb1* F (TGG AGC AAC ATG TGG AAC TCT) *Tgfb1* R (CCT GTA TTC CGT CTC CTT GGT), *Emr1* F (TAG TGG AGG AGG GCC AAT GT) and *Emr1* R (AAG GTG GGA CCA CAG AGA GT).

Measurement of inositol phosphate (IP) formation

The determination of IP formation was done as described in [1]. Briefly, HEK293 cells stably transfected with the human Calcium Sensing Receptor (CaSR) were labeled with [^{3}H]-inositol in serum-free medium for 24 h. Then cells were washed with mHBS (130 mM NaCl, 5.4 mM KCl, 0.5 mM $CaCl_2$, 0.9 mM $MgSO_4$, 10 mM glucose and 20 mM HEPES pH 7.4) and incubated at 37°C with or without lithium in order to block the activity of inositol monophosphatase activity. Urate at different concentrations was added and incubation continued for 20 minutes. Finally cells were extracted with 10 mM of ice-cooled formic acid and IPs formation was measured by anion exchange chromatography.

Full GLUT9 KO

Mice with systemic deletion of GLUT9 used for the characterization of the GLUT9 antibody are described in [2].

Supplementary data

Fig. S1 *Validation of rabbit mGLUT9 antibody*

Immunostaining control of GLUT9 antibody showed signals in kidney slices of WT animals (a) and no immunoreactivity in full GLUT9 KO kidney slices (b). (Scale bar: 50 μm). GLUT9 antibody recognized both GLUT9 isoforms in COS cells transfected with GLUT9a (c) or GLUT9b (d). No staining was obtained when cells are not transfected (e) (Scale bar: 25 μm). GLUT9 antibody detected both GLUT9 isoforms by Western Blot in HEK transfected with GLUT9a or GLUT9b, or not transfected (f). In kidney and liver, GLUT9 antibody recognize specifically

GLUT9 protein as no signal was observed with tissues obtained from full GLUT9 KO mice (fKO) (g).

Fig. S2 *Doxycycline effect on Glut9 expression in the kidney*

PCR on cDNA extracted from the kidney after 0, 6 and 14 days of doxycycline induction on control and triple transgenic mice. *Glut9a* and *b* showed recombination 6 days after the beginning of the induction. No recombination is detected in control mice (n = 3).

Fig. S3 *Doxycycline effect on Glut9 expression in the liver*

a. PCR on cDNA obtained from liver of control and triple transgenic mice treated during 0, 6 and 14 days with doxycycline, and 4 months after doxycycline induction. A recombination of both *Glut9a* and *Glut9b* is observed after 6 and 14 days of induction (n = 3).

b. Relative abundance of *Glut9* in liver 4 months after doxycycline induction measured by quantitative real-time PCR on cDNA from control and kiKO mice. Values are means ± SD relative to *Actb* (n = 5, * $P < 0.05$).

c. Glut9 protein expression in the liver 4 months after doxycycline induction in male and female mice (n = 2 to 6).

Fig. S4 *Doxycycline effect on Glut9 expression in the intestine*

Relative abundance of *Glut9* in ileum (a) and in colon (b) 4 months after doxycycline induction measured by quantitative real-time PCR on cDNA from control and kiKO mice. Values are means ± SD relative to *Actb* (n = 5).

Fig. S5 *kiKO mice have normal kidney anatomy and histology*

a. Control of the effect of doxycycline induction on sodium excretion. The sodium over creatinine ratio is not altered by the treatment with doxycycline. Values are means ± SD (n = 6, * $P < 0.05$).

b. Kidney macro-anatomies of control and kiKO mice are comparable.

c. Histology of the kidney observed with hematoxylin – eosin staining present no difference between control and kiKO mice.

Fig. S6 *kiKO mice have no kidney fibrosis or inflammation*

a. No difference in the mRNA expression level of fibrosis (*Vimentin*, *Fibronectin*, *Col1a1* and *Col3a1*) and inflammation markers (*Tgfb1*, *Emr1*) is detected between control and kiKO mice by qPCR. Values are means ± SD relative to *Actb* (n = 6).

b. Histology of the kidney observed with Masson's trichrome staining present no difference between control and kiKO mice (scale bar: 1mm).

Fig. S7 *kiKO mice have the same Aqp2 expression after water deprivation than control mice*

After 23h water deprivation, a significant increase of *Aqp2* expression compared to baseline was observed both in the cortex and the medulla part of the kidney, but no difference between control and kiKO was detected. Values are means ± SD relative to *Actb* (n = 6, * $P < 0.05$ compared to baseline in cortex and medulla).

Fig. S8 *Influence of urate on calcium-induced IP formation in human CaSR transfected cells.*

No difference can be detected after addition of 1, 3 and 5 mM of urate. Values are means ± SD (n = 3, * $P < 0.05$ compared to 1 mM Li^{+}, 0.5 mM Ca^{2+}).

Supplementary references

1. Mailland M, Waelchli R, Ruat M, Boddeke HG, Seuwen K (1997) Stimulation of cell proliferation by calcium and a calcimimetic compound. Endocrinology 138:3601-5 DOI 10.1210/endo.138.9.5417

2. Preitner F, Bonny O, Laverriere A, Rotman S, Firsov D, Da Costa A, Metref S, Thorens B (2009) Glut9 is a major regulator of urate homeostasis and its genetic inactivation induces hyperuricosuria and urate nephropathy. Proc Natl Acad Sci U S A 106:15501-6 DOI 10.1073/pnas.0904411106

Figure S1

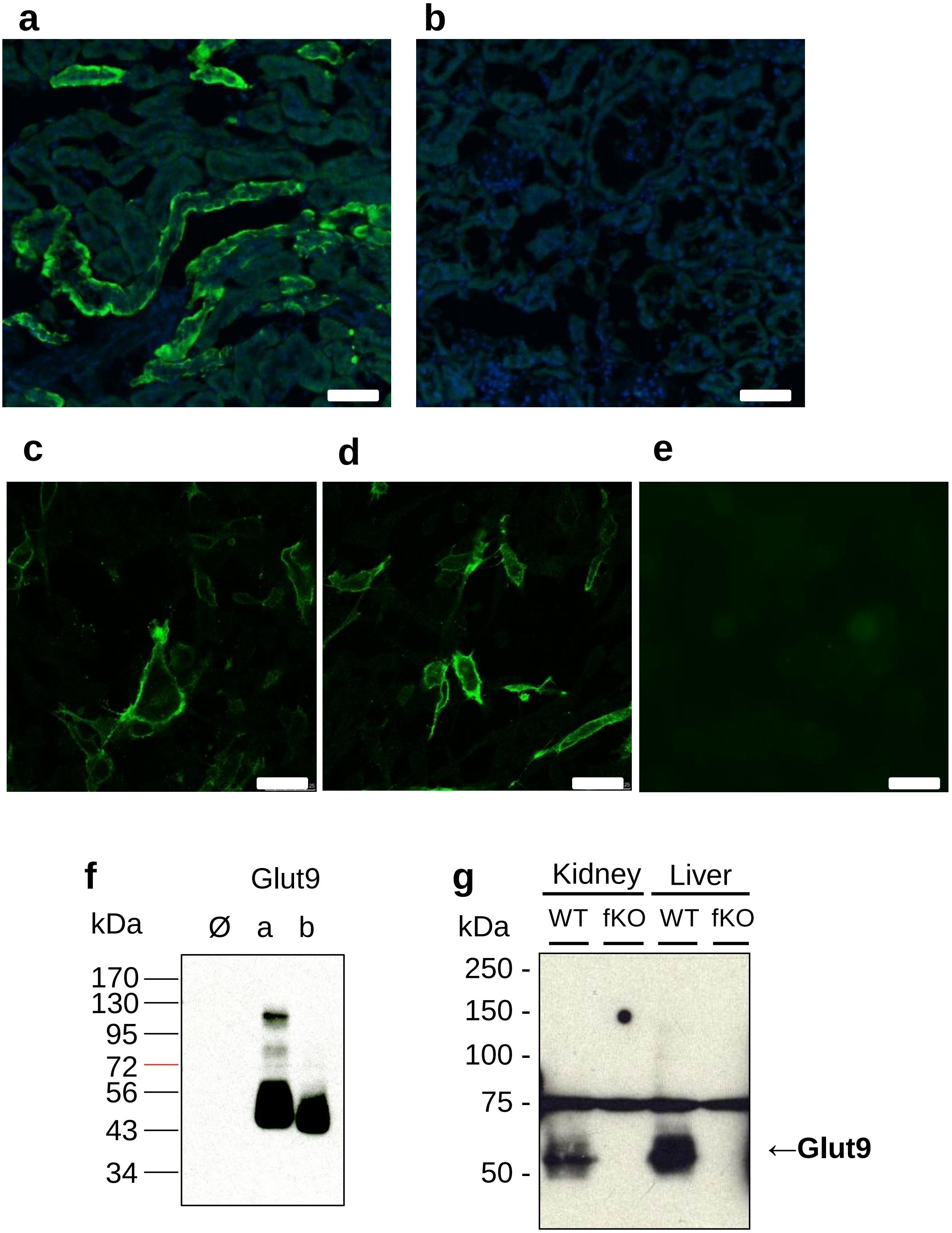
a
b
c
d
e
f
Glut9
kDa
Ø a b
170
130
95
72
56
43
34
g
Kidney
Liver
WT fKO WT fKO
kDa
250 -
150 -
100 -
75 -
50 -
←Glut9

Figure S2

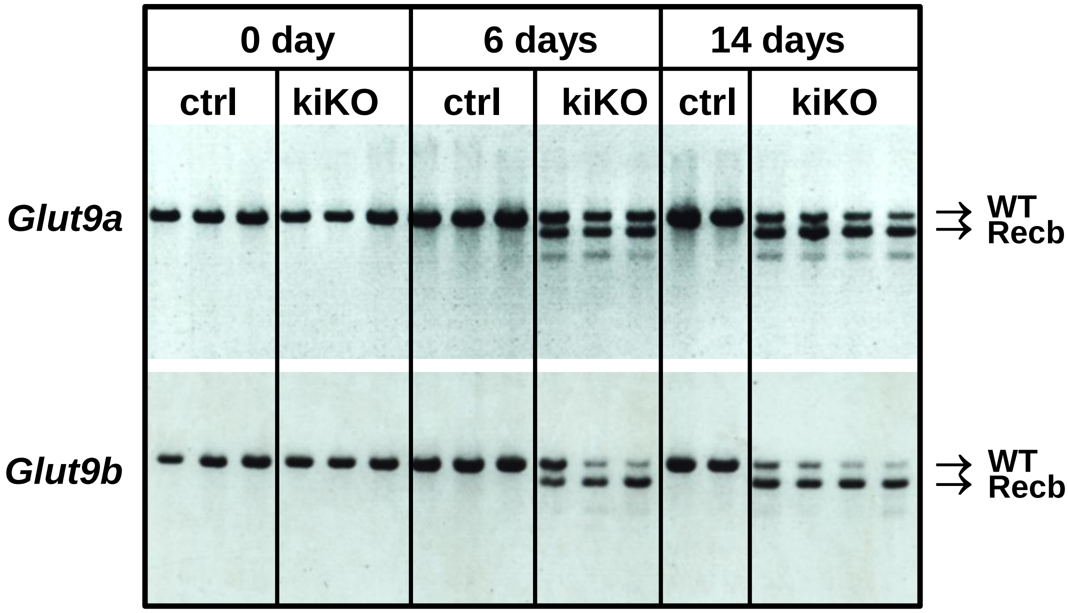
0 day
6 days
14 days
ctrl
kiKO
ctrl
kiKO
ctrl
kiKO
Glut9a
Glut9b
WT
Recb
WT
Recb

Figure S3

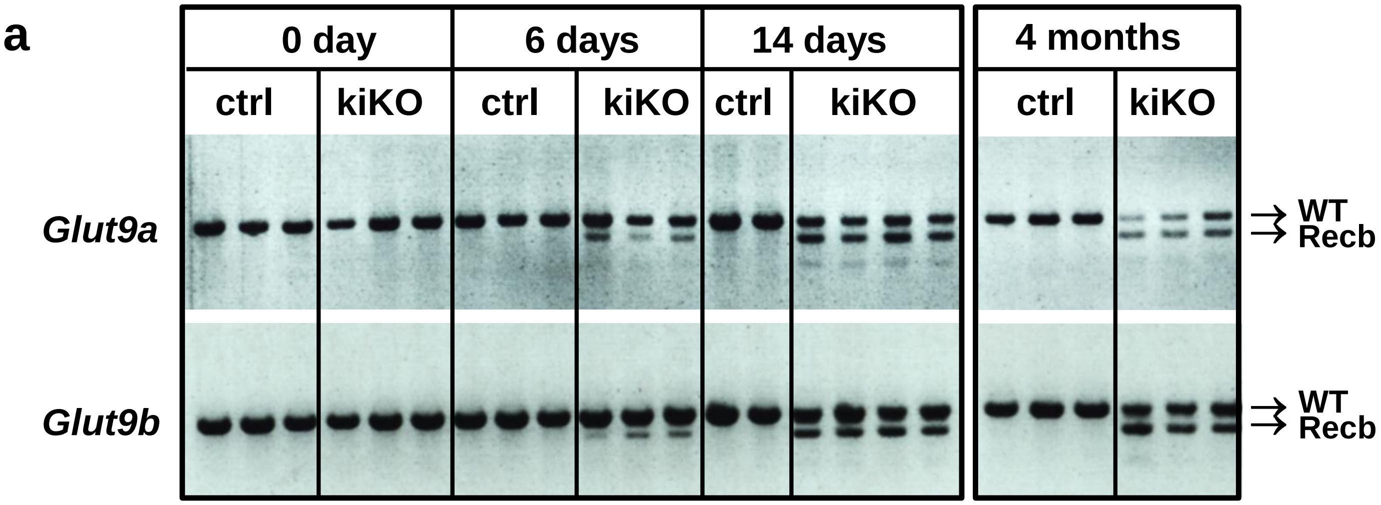

a
0 day
6 days
14 days
4 months
ctrl
kiKO
ctrl
kiKO
ctrl
kiKO
ctrl
kiKO
Glut9a
Glut9b
WT
Recb
WT
Recb

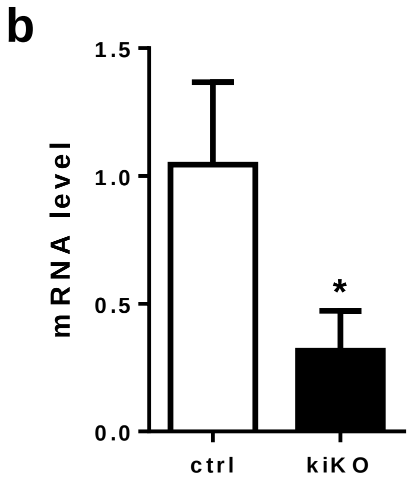

b
mRNA level
1.5
1.0
0.5
0.0
*
ctrl
kiKO

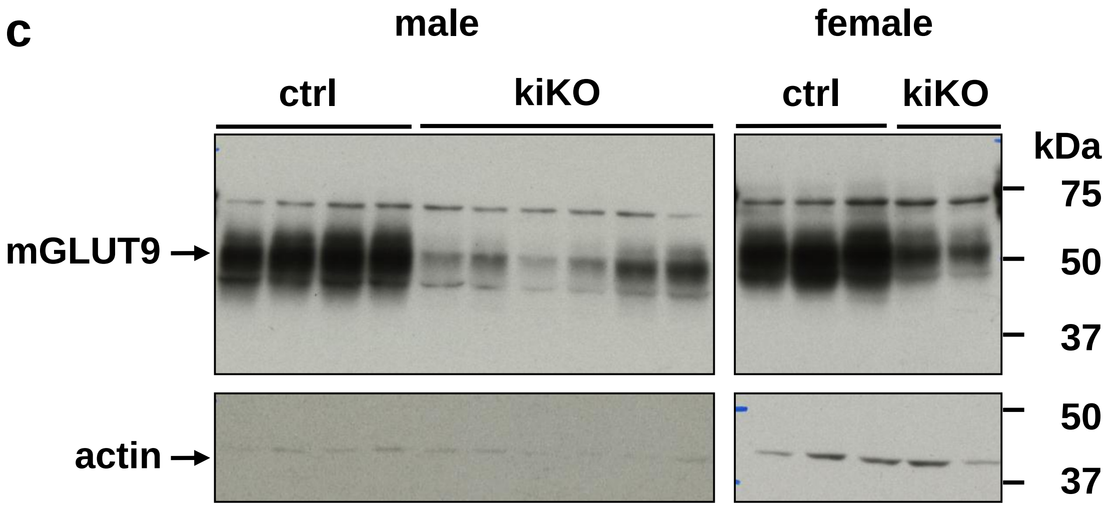

c
male
female
ctrl
kiKO
ctrl
kiKO
kDa
75
50
37
mGLUT9
actin
50
37

Figure S4

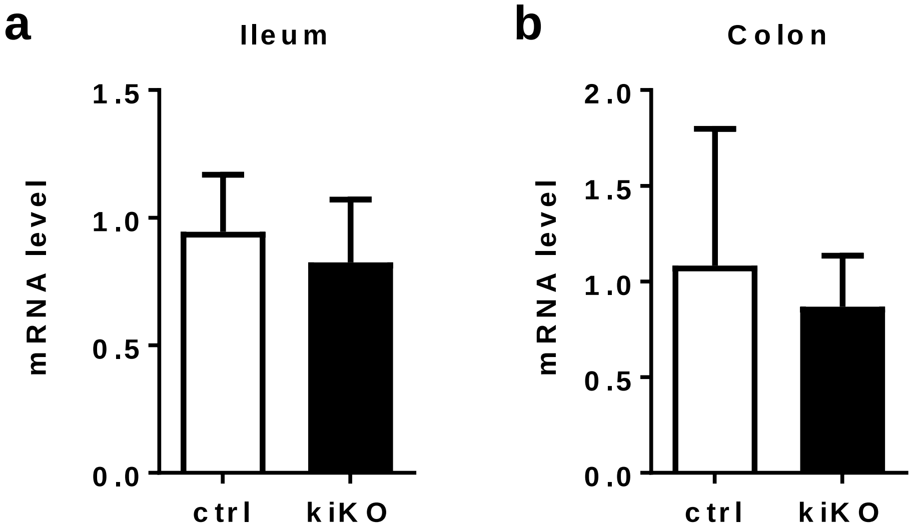
a
Ileum
mRNA level
1.5
1.0
0.5
0.0
ctrl
kiKO
b
Colon
mRNA level
2.0
1.5
1.0
0.5
0.0
ctrl
kiKO

Figure S5

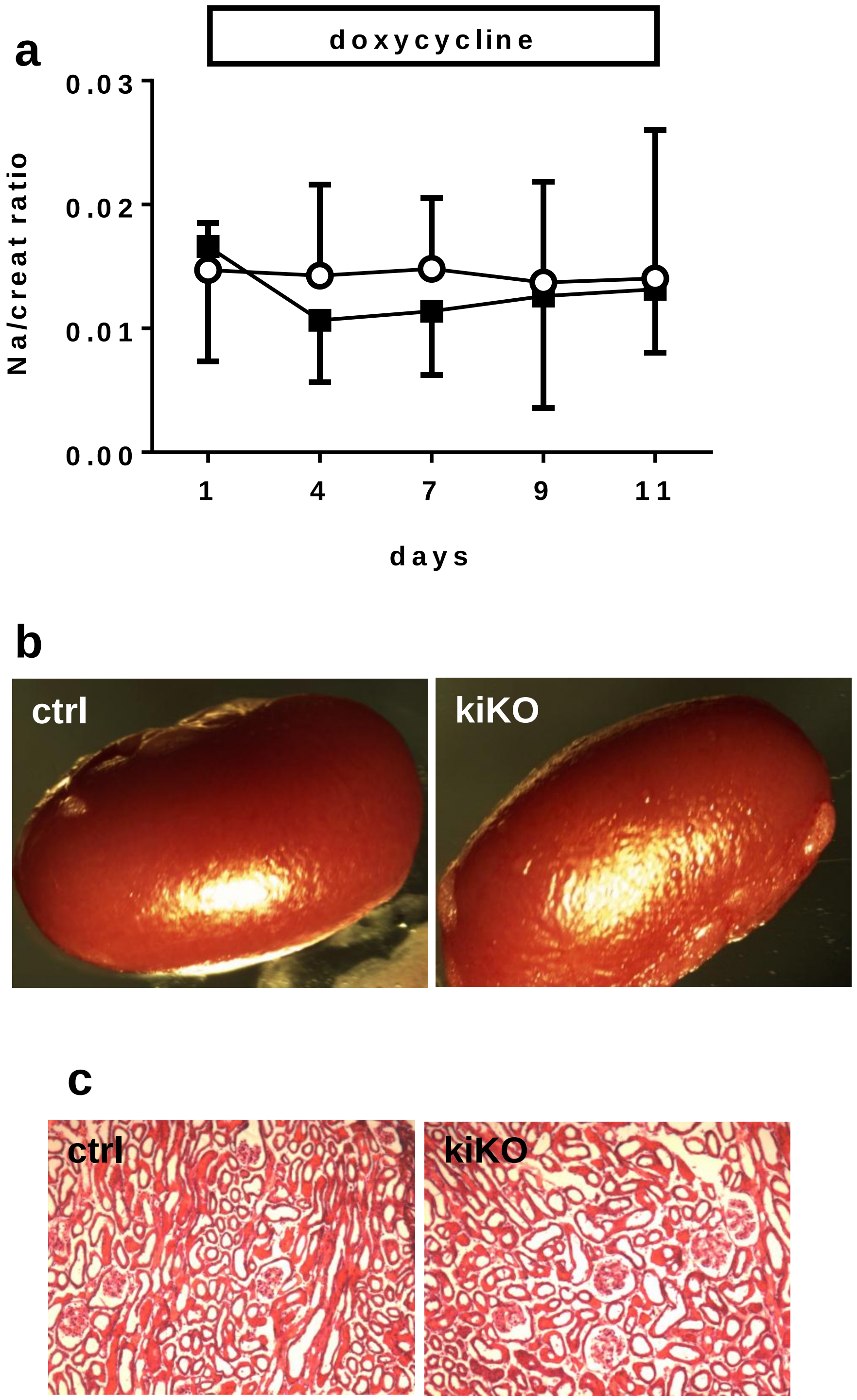

a
doxycycline
0.03
0.02
0.01
0.00
Na/creat ratio
1
4
7
9
11
days
b
ctrl
kiKO
c
ctrl
kiKO

Figure S6

a

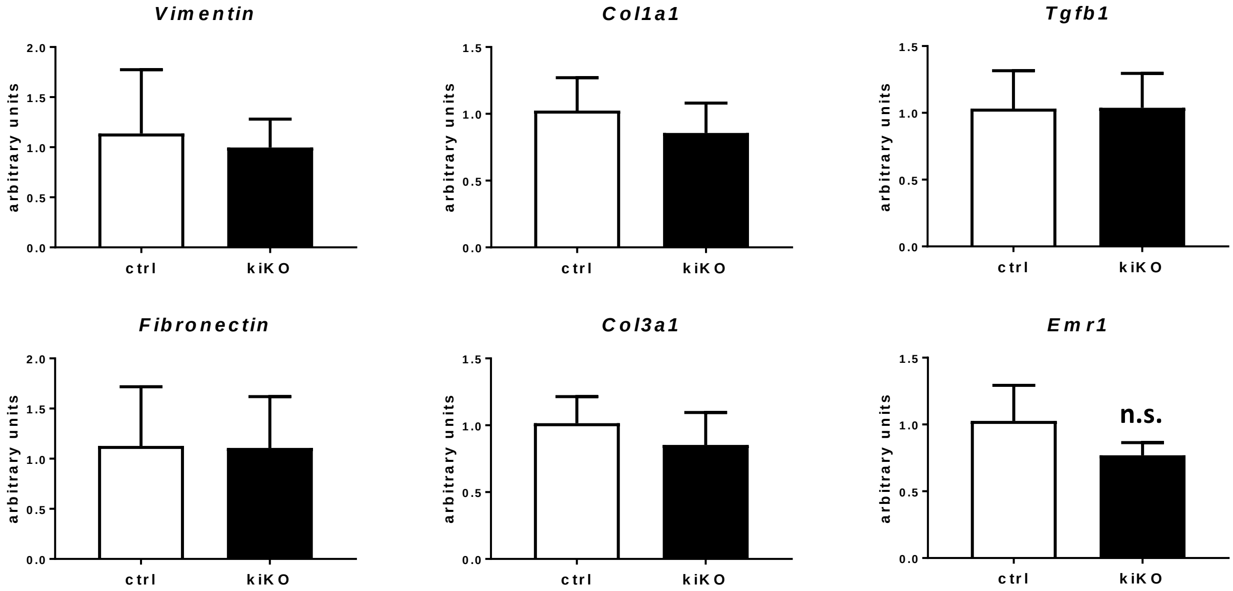

Vimentin
Col1a1
Tgfb1
Fibronectin
Col3a1
Emr1
arbitrary units
2.0
1.5
1.0
0.5
0.0
ctrl
kiKO
n.s.

b

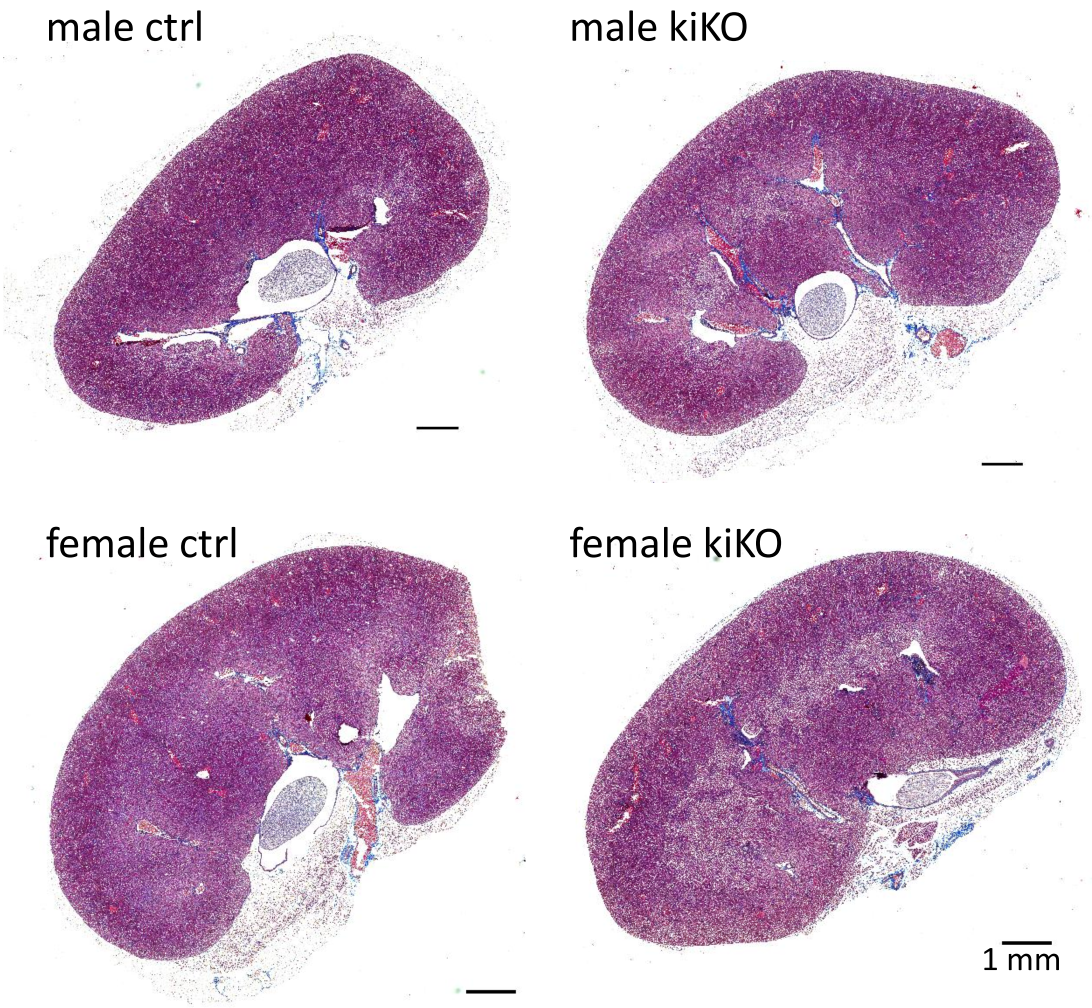

male ctrl
male kiKO
female ctrl
female kiKO
1 mm

Figure S7

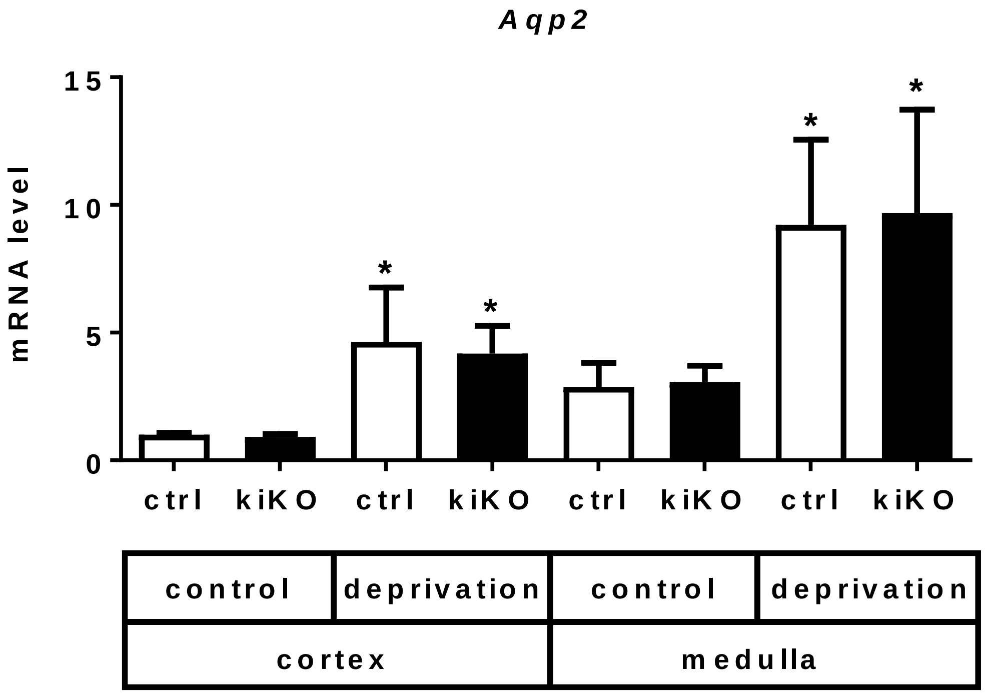

Aqp2
mRNA level
15
10
5
0
*
*
*
*
ctrl
kiKO
ctrl
kiKO
ctrl
kiKO
ctrl
kiKO
control
deprivation
control
deprivation
cortex
medulla

Figure S8

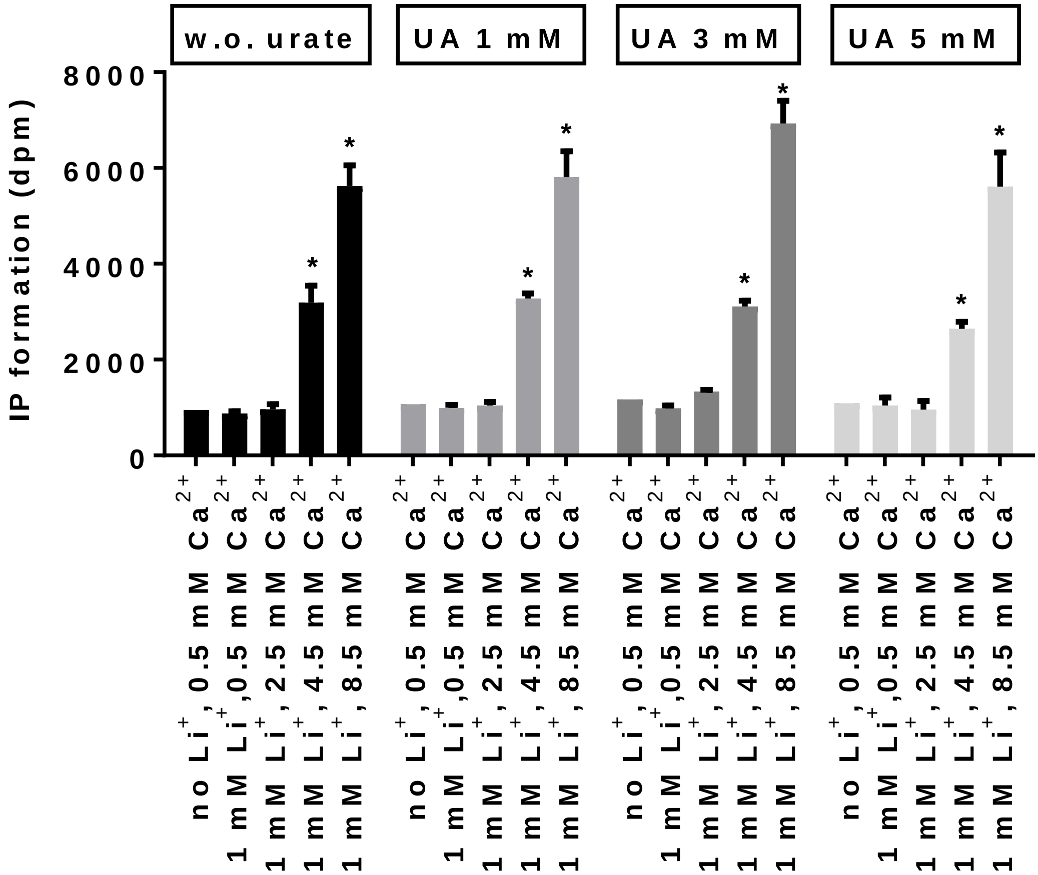

w.o. urate
UA 1 mM
UA 3 mM
UA 5 mM
IP formation (dpm)
8000
6000
4000
2000
0
*
no Li+, 0.5 mM Ca2+
1 mM Li+, 0.5 mM Ca2+
1 mM Li+, 2.5 mM Ca2+
1 mM Li+, 4.5 mM Ca2+
1 mM Li+, 8.5 mM Ca2+
